# The Global Health Network and globalization of higher education

**DOI:** 10.2196/jmir.1.suppl1.e5

**Published:** 1999-09-19

**Authors:** Ron LaPorte

**Affiliations:** ^1^University of Pittsburgh, School of Public HealthUSA

## Abstract

The year 2001 and the next millennium will soon be upon us. The major gains in health in the 20th century were primarily the result of improvements in public health including sanitation and immunization. Global health improvements will occur in the 21st century through improvements in information (in particular health training). We will describe a new paradigm for transnational training, the supercourse. In the next century global lecture-shareware training will take place, with Deming based quality control systems on the Internet. Faculty will thus share their best, most passionate lectures on the internet.

During the past 100 years there has been a 25-year increase in life expectancy. It has been estimated that 24 of the 25 years were the result of prevention. Most prevention activity is sharing of information. We are working with leaders from WHO, the World Bank, IBM, NASA, PAHO to create a discipline called telepreventive medicine. This is the application of low band with information systems (the Internet) to large numbers of well people to prevent disease. One of the most important aspect of this work is the establishment globalisation of prevention education; the Supercourse.

Question: What is the best way to improve health training/research?

Answer: Improve the lectures.

Question: How do we improve health training/research lectures:

Answer: Have academic faculty worldwide share their lectures:

Question: Will faculty share lectures?

Answer: Yes, The Supercourse has 1107 faculty from 101 countries who created a Library of Lectures with 110 lectures on the Internet with quality control, and cutting edge cognitive design. This is being shared worldwide.

We are developing a "Library of Lectures" with passionate lectures in public health from across the world such as seen here from South Africa. We propose to expand this to all areas of research in health. Our program consists of:

1. Shareware: A Global faculty is developing and sharing their best, most passionate lectures. This benefits all. The experienced faculty member can beef up their lectures that are not cutting edge. New instructors reduce preparation time and improve their lectures, as they can employ state of the art lectures from others. Faculty in developing countries have access to current public health information for the first time. The concept is that of a library of lectures for all to use is in many ways similar to that of "shareware" on the computer.
2. Statistical Quality Assurance: We have established a Deming Model of statistical quality control to monitor lectures over time
3. Supporting the teachers: The Library of Lectures consists of exciting lectures by public health experts in the field. The classroom teacher "takes" them out for free like a library book. There is no direct teaching of students from a distance, rather the concept of the system is to provide cutting edge material for all faculty to present.
4. Hypertext comic book: The lectures are icon driven, and the students can go deep into the Internet for more information through hyperlinks. It is based upon PowerPoint for ease of usage
5. Presentation Speed: We have discovered technologies to speed access to lectures world wide
6. Text books: The British Medical Association has put 2 current text books on line for us
7. Multilingual: For global use, this must be multilingual, the first lecture is in 8 languages
8. Voice-Sound Video: We are using state of the art Internet voice-video systems. We soon will be using "clickable" voice video

We have published over 68 papers in leading medical journals including the Lancet, British Medical Journal, Nature Medicine among others. We are working with PAHO to put mirrored servers into every medical school in the Americas this year, with 5 years we should reach globally all medical schools. WHO has developed a Supercourse.

Initial pilot studies reveal that 2500 individuals will see each lecture each year, which is 50 times that of our classroom teaching. We have beta tested lectures in 2 centers in Japan and one in South Africa with very positive results. We are now developing a Chinese Heritage course.

